# Effects of low-intensity secondary theory-based Chatbot intervention for adolescents with digital addictions: protocol for a randomized controlled trial

**DOI:** 10.1186/s12888-026-08375-5

**Published:** 2026-07-27

**Authors:** Xue Yang, Xin Wang, Nick Ka-wo Tse, Zixin Wang, Winnie W. S. Mak, Phoenix Kit-han Mo, Samuel Yeung-shan Wong, Qingpeng Zhang, Liping Zhang

**Affiliations:** 1 The Jockey Club School of Public Health and Primary Care, Faculty of Medicine, The Chinese University of Hong Kong, Hong Kong, Hong Kong Special Administrative Region China; 2 HKCT Institute of Higher Education, Hong Kong, Hong Kong Special Administrative Region China; 3 Department of Psychology, The Chinese University of Hong Kong, Hong Kong, Hong Kong Special Administrative Region China; 4 Department of Pharmacology and Pharmacy, Li Ka Shing Faculty of Medicine, The University of Hong Kong, Hong Kong, Hong Kong Special Administrative Region China; 5 Harvard Medical School, Harvard University, Boston, USA

**Keywords:** Adolescent, Chatbot, Internet gaming disorder, Social media addiction, Randomized controlled trial, Motivation interviewing

## Abstract

**Background:**

Adolescence is a unique and vulnerable stage, and mental health problems and digital addictions such as internet gaming disorder (IGD) and social media addiction (SMA) are arising among adolescents. The current study thus aims to develop and evaluate the effectiveness of a secondary low-intensity stage of change (SoC)- and motivation interviewing (MI)-based Chatbot-assisted intervention in reducing common digital addictions, sedentary lifestyles, and mental distress, compared to an active control group. We also examined whether the treatment effects are mediated by the perceived autonomy and competence.

**Methods:**

The proposed study is a two-arm randomized controlled trial (RCT) comparing a five-week low-intensity SoC and MI-based Chatbot-assisted group with a psycho-educational control group both via WhatsApp. The expected number of participants with clinical digital addiction symptoms is 330. The primary outcome is the composite score of IGD and SMA, while the secondary outcomes include sedentary lifestyle, quality of life, psychological distress, and eHealth literacy. Perceived autonomy and competence are set as mediators. The evaluation involves baseline evaluation (T0), post-program evaluation (T1), and a 3-month follow-up evaluation (T2). In addition to the psycho-educational materials received by the control group, the intervention group will receive the SoC- and MI-based chatbot intervention via WhatsApp.

**Discussion:**

This proposed RCT study may fill critical knowledge and service gaps by evaluating an evidence-based and Chatbot-assisted intervention program for at-risk adolescents. By applying an active control with psycho-educational materials, the current study may determine whether digital interventions offer a more cost-effective and scalable alternative to traditional information-based methods. The findings may provide a scalable and sustainable intervention for adolescents with common digital addictions and mental health problems, especially in resource-limited settings.

**Trial registration:**

The RCT protocol was pre-registered at the ClinicalTrials.gov registry (NCT06821373) on February 09, 2025.

**Supplementary Information:**

The online version contains supplementary material available at https://doi.org/10.1186/s12888-026-08375-5.

## Introduction

### Adolescent common digital addictions

According to World Health Organization (WHO), adolescence is the phase of life between childhood and adulthood, from ages 10 to 19, which is a unique stage of human development and an important time for laying the foundations of good health and healthy lifestyles. WHO also highlights that adolescents can experience devastating behavioral and mental health issues, such as addiction to video games, depression, anxiety, and suicide. Digital platforms, video games, and social media (e.g., TikTok, Instagram) in particular, can be highly addictive and cause or exacerbate sedentary lifestyles, physical inactivity, mental distress (depression, anxiety, sleep problems, suicide), other health problems (e.g., attention deficit disorder, eating disorders, body dysmorphia disorder, myopia) in adolescents [1]. A meta-analysis suggested that prolonged screen time and sedentary lifestyle are emerging risk factors of chronic disease in adolescents [2]. Especially, Internet gaming disorder (IGD) has been defined as a mental disorder by WHO due to its harms to health and the brain in similar ways to substance abuse [3]. IGD and Social Media Addiction (SMA) are the most prevalent digital addictions in adolescents, compared to addiction to Internet gambling/shopping/pornography. Previous studies have shown a significant prevalence of IGD (8.8% to 10.4%) [4] and SMA (14% to 31%) [5]. A survey in a representative sample of Hong Kong (HK) adolescents found that about 15% of adolescents were classified as having IGD [6]. The average scree time was 2.45 (SD = 2.32) hours/day in adolescents, but hours for outdoor exercise were only 0.64 (SD = 0.70) [7]. However, evidence-based treatment for common digital addictions is lacking. Recent systematic reviews and meta-analyses [8–10] only identified five randomized controlled trials (RCTs) targeting adolescent IGD, which were mainly cognitive behavioral therapy (CBT)-based; only one RCT [11] and one non-RCT [12] for adolescent SMA were identified and both were conducted in Thailand.

### Importance of adolescent eHealth interventions

Although digital platforms may increase behavioral/mental problems, they can be beneficial by facilitating eHealth interventions for these problems [13]. eHealth interventions are particularly useful within school settings as they are cost-effective, reduce stigmatization, and can be easily disseminated to larger groups of students. COVID-19 also posed a grim challenge to monitoring and intervening in students’ mental/behavioral problems for school social workers and counsellors, making eHealth interventions essential. A recent meta-analysis concluded that eHealth school-based interventions were effective on fruit/vegetable intake, physical activity, and screen time, and highlighted that interventions for digital use behaviors (screen time) are relatively lacking [2].

### Lack of evidence-based Chatbot-assisted eHealth interventions in adolescents

Chatbots are emerging as a digital solution for psychological/behavioral interventions through human-like communication while maintaining anonymity (especially for sensitive conditions like addiction) and minimizing healthcare resource [14]. Chatbot is a rule-based or artificial intelligence-based communication software that uses a mobile device to provide answers and relevant information in response to questions posed through text or voice conversations. Chatbots have the potential to enhance clients’ engagement, reduce costs, refine workflow efficiency, and monitor and improve health outcomes [14, 15]. Because Chatbots are optimized for mobile devices and can thus obtain the necessary information without the need to install a separate app, the number of users and the service area are increasing significantly.

Chatbots have been applied to motivate and sustain behavioral changes and reduce addictions, such as gambling [16] and substance use [17]; Two were conducted in adolescents by the same team and they were non-RCTs [18, 19]. A systematic review and meta-analysis of chatbot‐delivered interventions for mental health promotion among young people included 11 RCT studies and found that Chatbot-delivered interventions significantly reduced psychological distress among young people [20]. A pilot RCT (*n* = 15) using text-based healthcare Chatbots (THCB) for children’s obesity found that patients demonstrated strong intervention adherence and a high level of interaction with the peer-roled THCB [21].

### Stage of change (SoC) model of addictive behaviors

The SoC model has been widely used in chatbot interventions for behavioral changes [22, 23]. The SoC construct grounded on the Trans-Theoretical Model (TTM) refers to qualitatively different psychologic and behavioral states in the process of behavior change. The TTM conceptualizes behavioral change as a transition through five stages: Pre-contemplation (PC), Contemplation (C), Preparation (P), Action (A), and Maintenance (M). Each stage of change requires different information needs and skills and approaches to advance to the next stage. Individuals may also drop back or jump over stages. Such “recycling” is a common observation in behavior change, especially for addictive behaviors. Tailored interventions based on SoC have been effective for addictive behaviors, including smoking, drinking, and eating disorders [24]. One pilot (non-RCT) SoC-based intervention combined with Motivation interviewing (MI) among 59 adolescents reported significant pretest-posttest changes in Internet addiction [25].

### Motivation interviewing (MI) for addictive behaviors

SoC is recommended to combine with MI for tailored/personalized interventions. Motivation plays a vital role in an individual’s decision to change at each stage and facilitates the transition through the five stages. A recent review has identified that promoting intrinsic and self-regulated motivation is required to reduce behavioral problems; it stems from satisfaction of one’s autonomy and competence [26]. It is a client-centered, non-directive, goal-oriented counselling technique that helps clients to review the pros and cons of change and to identify their internal motivation for change [27]. MI is grounded on four principles to express empathy to the client, help client to recognize pros and cons of the behavior, and enhance self-efficacy [27]. Evidence has shown the effectiveness of Internet-based brief MI for adolescent substance-use addiction [28], physical activity, screen time, and nutrition [29–31]. No RCT for Internet-based MI was identified for adolescent IGD or SMA.

### Research gaps

1. There is lack of theory-based low-intensity intervention for adolescent common digital addictions. We only identified five RCTs for adolescent IGD and one for adolescent SMA with small sample sizes.
2. Chatbot is an emerging and promising solution for mental/behavioral health promotion especially in the context of lack of resource of mental healthcare. We did not identify any RCT for adolescents or digital addictions, although Chatbots have shown emerging evidence for substance-use addictive behaviors and adolescent behavioral health. In addition, online brief MI or SoC-based RCTs for adolescent IGD or SMA are lacking, although both therapies/theories have shown evidence on their effectiveness to reduce screen time or substance-use addictive behaviors. Chatbot interventions based on MI and SoC have been emerging, but lack RCT studies.
3. Little is known regarding the potential mechanisms between chatbot interventions and behavioral changes. According to Ryan and Deci’s Self-Determination Theory, autonomy and competence are two basic psychological needs and such needs’ satisfaction is closely related to adolescents’ current and future behavioral and mental health as adolescence is an important stage of cognitive, behavioral, and social maturation. MI facilitates elicitation of clients’ self-endorsed reasons for change, which mirrors satisfaction of autonomy; it also affirms client’s strengths and previous successful attempts, leading to satisfaction of the needs of competence. Thus, MI may nurture the perceptions of autonomy and competence; in turn, such perceptions can enable individuals with ambivalence and weak intention to endorse a more self-endorsed motivation to change [27]. On the other hand, SoC-based Chatbots can provide immediate and personalized monitoring and advice on coping and self-regulation strategies regarding different stages of change, which may enhance clients’ self-efficacy and perceived competence of behavioral/self-control; in addition, such services are self-directed [32]. Consequently, competence and autonomy may be enhanced and play as potential mediators between Chatbot-facilitated intervention and behavioral change. Therefore, we propose and test the mediation effects of perceived autonomy and competence (Appendix 1).

### Aims and hypotheses

This project aims to develop and evaluate the effect of a secondary low-intensity SoC and MI-based Chatbot-assisted intervention on reducing common digital addictions (composite outcome of IGD and SMA [primary outcome]), as well as sedentary lifestyles (including entertainment screen time) and mental distress (secondary outcomes) in at-risk adolescents by conducting a two-arm RCT (intervention group versus psycho-educational control group).

#### Hypothesis 1

The level of common digital addictions is lower in intervention group compared to control group at post-test and 3-month follow-up.

#### Hypothesis 2

Health status (sedentary lifestyles, quality of life, mental distress, eHealth literacy) is significantly better in intervention group compared to control group at post-test and 3-month follow-up.

#### Hypothesis 3

The observed between-group differences in the behavioral and mental outcomes are mediated by changes in perceived autonomy and competence.

## Methods

### Study design

The proposed study is a two-arm RCT comparing a five-week low-intensity SoC and MI-based Chatbot-assisted group with a psycho-educational control group both via WhatsApp. Baseline evaluation (T0), post-program evaluation (T1), and 3-month follow-up evaluation (T2) will be conducted. The intervention is a tailored secondary intervention, which targets the high-risk group based on the IGD/SMA screening results. Our participants include adolescents aged 10–19 (i.e., grade 5–12 students). We will invite 8 primary schools and 8 secondary schools to join the screening. We expect to recruit at least 330 students for the RCT. Inclusion criteria are: (1) students at grade 5–6 in primary schools or grade 7–12 in secondary schools, (2) positive screening results of IGD and/or SMA) based on the validated screening tools, (3) using smartphone or Internet on daily base, (4) students’ and parental consent, and [5] Chinese speaking. The exclusion criterion is the current use of any psychotropic medication.

### Screening tools

IGD symptoms are assessed using the Nine-Item Internet Gaming Disorder Scale–Short Form (IGDS9-SF), which was developed based on the DSM-5 criteria [33]. It consists of nine questions that assess IGD symptoms, including preoccupation, tolerance, withdrawal, unsuccessful attempts to limit gaming, deception or lies about gaming, loss of interest in other activities, use despite knowledge of harm, use for escape or relief of negative mood, and harm. The response options for each item include “yes = 1” and “no = 0”. The overall score of the scale ranges from 0 to 9, with a higher score implying a higher level of IGD symptoms. Participants with score ≥ 5 will be classified as having probable IGD. The scale was validated among Chinese adolescents [34].

SMA symptoms are measured by the 6-item Bergen Social Media Addiction Scale (BSMAS) developed [35]. The items are rated using a Likert type scale ranging between 1 (very rarely) and 5 (very often). A higher sum score in the BSMAS indicates a greater likelihood of being at risk of developing social media addiction. A cut-off score over 19 indicates probable SMA [36]. The Chinese version was demonstrated to have good psychometric properties [37].

### Primary outcome

Composite outcome of IGD score and SMA score: we define a simple composite as the sum of the two outcomes. Because each outcome measure uses a different scale and has different statistical properties, we will standardize the two variables (i.e., scores of IGD and SMA) first and then combine the two standardized scores of the outcomes into a composite [38].

### Secondary outcomes

Sedentary lifestyle is defined as any waking behaviors such as sitting or leaning with an energy expenditure of 1.5 metabolic equivalent task (MET) or less [39]. Sedentary lifestyles will be measured by the 10-item sedentary lifestyles questionnaire (SLQ), which has been used in Chinese school children [7]. Subjects will report the average daily time spent (hours) during the weekdays and weekends in the following activities: Using smartphone or tablet PC for learning/ Doing paper homework or reading or writing/ sedentary leisure time without time for screen leisure (e.g., drawing and playing chess) [7]. Average number of daily hours will be calculated as (total sedentary weekday hours × 5 + total sedentary weekend hours × 2) ÷ 7.

Quality of life is measured by the EQ-5D-Y scale (Y for youth) [40]. The EQ-5D-Y uses a similar 5-dimenional descriptive system with the EQ-5D but child-friendly wording, referring to mobility (‘walking about’), self-care (‘looking after myself’), usual activities (‘doing usual activities’), pain and discomfort (‘having pain or discomfort’), and anxiety and depression (‘feeling worried, sad or unhappy’). Each dimension includes one item which has three functioning levels: no problem, some problems, and a lot of problems. The EQ-5D-Y also includes an easily understandable modification of the vertical, graduated Visual Analogue Scale (VAS) of EQ-5D, where the respondent rates his or her overall health status on a scale from 0 to 100 with 0 representing the worst and 100 the best health state he or she can imagine. All items refer to the health state ‘today’. The scale has shown good reliability and validity in Chinese students [41].

Psychological distress is assessed by the Chinese version of K6 [42]. It comprises six questions that ask respondents to rate how frequently they have felt ‘nervous’, ‘hopeless’, ‘restless or fidgety’, ‘so depressed that nothing could cheer you up’, ‘that everything was an effort’, and ‘worthless’ during the past 30 days. Response options include 0 ‘none of the time’, 1 ‘a little of the time’, 2 ‘some of the time’, 3 ‘most of the time’, and 4 ‘all of the time’.

eHealth literacy is assessed by the 8-item eHealth Literacy Scale (eHEAL) [43], which was validated in Chinese student population [44]. The sample items include ‘I know how to find helpful resources on the internet’ and ‘I know how to use the internet to answer my questions about health’. Ratings are made on 5-point Likert scales, ranging from 1 = strongly disagree to 5 = strongly agree, with higher scores indicating a higher level of eHealth literacy.

### Mediators

To assess autonomy to reduce dependence on Internet gaming/social media, the subscale of the Psychological Need Satisfaction in Exercise Questionnaire (PNS-A) will be adapted and used [45]. The Chinese version was demonstrated to have good psychometric properties [46]. The scale includes six items measuring psychological need fulfilment of autonomy. Items are rated on a 6-point Likert scale, ranging from 1 (false) to 6 (true). Total scores range from 6 to 36, with higher scores being indicative of higher degrees of autonomy.

The 6-item competence subscale of PNS (PNS-C) will be used to assess competence (self-efficacy) to reduce dependence on Internet gaming/social media [45]. Total scores range from 6 to 36, with higher scores being indicative of higher degrees of autonomy or competence. Background information, including age, gender, grade, stressful life events, use of mental health services, school, residency, living arrangement, facilitator/avatar, perceived parental/peers’ screen time, and parental education levels, was recorded at baseline.

Process evaluation was conducted at pos-test to investigate participants’ satisfaction (e.g., entertainment, flow, trustworthiness, and overall appreciation scores) with the intervention.

### The control group

Participants will receive psycho-educational materials without any SoC or MI-based content via WhatsApp weekly for five weeks. Such psycho-educational interventions are widely used in behavioral/mental health promotion and have been demonstrated to be effective in increasing knowledge and awareness of a behavioral/mental problem. Based on a comprehensive literature review and our previous psycho-educational programs for behavioral health promotion, the research team will design the materials. Themes include definitions, factors, and health consequences of excessive digital use, IGD and SMA, information of healthy lifestyles, self-regulation skills, and help-seeking resources.

### The intervention group

#### Intervention contents

The intervention group will receive five modules based on the five stages of SoC delivered by a Chatbot (Appendix 2). The Chatbot will build up rapport with the participants, monitor their stage by a few simple questions based on previous SoC studies, and provide stage-tailored packages of response, advice, and feedback (one of the five modules) accordingly to enhance their motivation and self-efficacy each week. For those who click ‘yes’ to question assessing the first stage of change (PC), Chatbot will make a record and automatically ask questions assessing the next stage; for those who click ‘no’, Chatbot will automatically send them the module corresponding to their SoC via WhatsApp (Appendix 2). Each module will take 10–15 minutes. In total, it will take 1 hour to complete all the modules. To capture adolescents’ interest, all the advice will be in the form of videos, gamification, metaphors, daily examples, experiential exercises, or lively narrative stories. Videos will be animated and storytelling. To avoid participants receiving the identical package twice, five slightly different versions corresponding to each SoC will be prepared. In each session, Chatbots will randomly select one package for participants. Chatbots will also send weekly reminder messages automatically to reinforce their self-efficacy and respond to common asked questions instantly between week intervals. Trained MI research assistant/associate (RA) will also stand by to check and respond to questions regularly if any (per 12 hours).

### Intervention development

#### Protocol development

The PI (a health psychologist trained with MI and experienced in conducting interventions for adolescent mental and behavioral health) and co-Is (NT: a certified MI trainer and registered social worker who specializes in the dissemination of MI; WM: a clinical psychologist experienced in Chatbot-delivered mental health interventions; SW: a clinician experienced in mental health interventions; PM: a health psychologist experience in adolescent mental and behavioral health interventions) will develop the study protocol. The MI protocol is fairly consistent with the published MI Manuals. Certain elements of the protocol are adjusted to better fit the issues with which adolescent populations struggle. The same protocol is used with all participants, but as with any treatment study, adjustments are made to conform to each individual. Slight adjustments will also be made for the early, middle, and late adolescent groups. Focus group discussions in youth will be conducted to develop the protocol. The team will conduct a 2-week pilot run of ten youth and obtain their feedback to refine the contents.

#### Avatar design

The cartoon avatar (virtual/e-coach played by Chatbot) in all the videos and images of the intervention materials will be designed. The physical appearance of the avatar will be based on the results of a series of focus group discussions in youth that discuss how a motivating and reliable avatar (coach) should look. This consistency of the physical appearance throughout the intervention may help the participants to better engage in the intervention.

#### Chatbot design (Appendix 2)

Our Chatbots will be developed and maintained in collaboration with IT experts (our co-Is: LZ, QZ) on the WhatsApp platform because of their wide popularity in local adolescents and user-friendly interface for extensions. The Chatbot will use a mixture of techniques so that the human-Chatbot interactions will not merely be rule-based, but also learn from the evolving knowledge graph through machine learning. The same professional team will maintain the health status of the Chatbot during the intervention period. Monthly review of the Chatbot performance will be conducted by co-Is. A feedback loop is also incorporated in the Chatbot, users are invited to leave comments. Improvements will be made accordingly.

### Training and quality control of MI

#### MI trainees

The trainees include RAs (who will stand by and answer questions in case the participants would like to contact real Coach or require clarification) with degrees in psychology, social work, or counselling. The PI will supervise the MI interviewers together with NT.

#### Training workshop

Self-study will take place one week before the workshop; the trainees read a reference book *Motivational Interviewing: Helping People Change* (3rd Edition) to familiarize themselves with the concept and theory of MI. The 3-day workshop includes: (i) a recap of the theory and concept of MI (video demonstrated by world experts and discussion); (ii) skills-based training, where NT will demonstrate MI skills and lead skill-building practices for the trainees, such as modeling of specific skills and brief practice exercises; and (iii) a role-play practicum in which each trainee will conduct MI interviews via WhatsApp with youth who meet the inclusion criteria of the proposed study. Audio-taping will be made. The trainers will conduct coding to evaluate the performance and fulfillment of proper procedures. Trainees should attain either fair or good level of proficiency before they are qualified to deliver MI interviews. Otherwise, another round of MI, audio submission, coding, and feedback will be conducted.

#### Continuous supervision and support

The trainers and RAs will meet monthly to solve problems encountered and improve the performance of MI. Questions can also be asked between meetings.

#### Coding

Coding will be conducted at different phases to evaluate whether the MI processes fulfill the requirements (e.g., showing empathy). The Motivational Interviewing Treatment Integrity version 4.2.1 (MITI4.2.1) coding manual will be used for coding to evaluate such quality of MI. The two trainers are certified coders and will conduct the coding.

#### Fidelity checks

All videos will be formatted in a way that participants cannot fast-forward; the system will automatically record time between starting and leaving the video-links. The number of attended modules and random questions regarding the intervention content will be recorded to check participants’ engagement.

#### Procedures (Appendix 3 for flow chart)

### Invitation

We will send invitation letters and information sheets (logistics and benefits) of the project to all the primary and secondary schools in HK. To encourage and appreciate the schools’ participation, comic booklets (both children’s version and parental version) and small gifts (e.g., bookmarks) for healthy lifestyle promotion well designed by health psychologists (PI and Co-Is) will be distributed to all students and their parents of the participating schools after the study. Also, a school-based report including the screening results and professional advice at the school level (without individual information) will be provided to the schools.

### Screening and recruitment

The school-based screening survey will be conducted in classroom settings by research staff in the absence of teachers. It includes scales assessing IGD, SMA, and some background questions, and will take 10 min to complete. Assisted by teachers and school social workers, students with one or more unhealthy lifestyles will be invited to join the intervention study.

### Randomization and baseline

Eligible participants will be evenly randomized into the intervention or control groups, based on computer-generated random number and block randomization (size of four). Randomization using envelopes to assign participants to a treatment group will be used in order to conceal the allocation sequence. We will perform stratified randomization by forms (grade 5–6, grade 7–9, grade 10–12) to guarantee intervention group and control group have similar proportion of early, middle, and late adolescents. The randomization will be clearly explained to the students and parents and in the information sheet. Baseline evaluation will be conducted prior to commencement of the first intervention session.

### Ethics

Data confidentiality was strictly maintained. Participants were assured that their data would be only used by the researchers and remain inaccessible to third parties, including teachers and parents. They were also informed that participation was voluntary and refusals had no consequences. The study was conducted in accordance with the Declaration of Helsinki. Ethics approval was obtained from the Survey and Behavioural Ethics Committee of the corresponding author’s university (Ref.#2022.589). All participants and their parents provided written informed consent.

### Sample size

We use the continuous variable of IGD and SMA as the primary outcome for sample size planning. There was no Chatbot, SoC, or MI-based intervention for IGD or SMA. Previous research showed that the effect size of MI-based treatment for screen-time ranged from Cohen’s d = 0.31 to 0.80 [11, 29, 47, 48]. Most of the related studies reported large effect sizes [29, 47, 48]. We assume the effect size is at a medium level (Cohen’s d = 0.50) in current study. Using G*Power 3.1, it is estimated that the sample size of 64 (128 in total) will allow us to detect a between-group difference (intervention group versus control group) at a minimum effect size (alpha = 0.05, power = 0.8, 2-sided independent t-test). For Structural Equation Modeling (SEM) to test the mediation effects, a widely accepted rule of thumb is 10 cases/observations per indicator variable in setting a lower bound of an adequate sample size [49]. The sample size of 50 will be adequate (1 indicator for intervention group, 1 for perceived autonomy, 1 for competence, 1 for outcome at post-test, and 1 for outcome at follow-up).

### Data processing and analysis

The study will be evaluated in agreement with the CONSORT statement. The baseline characteristics of intervention group and control group will be represented. Between-group differences in primary and secondary outcomes will be compared by Student’s t-test. Generalized Linear Mixed Models (GLMM) will be used to detect the between-group differences in the continuous variables of primary outcomes and secondary outcomes across time points (T0, T1, T2). The potential mediators (autonomy and competence) will be tested by using SEM and bootstrapping analysis (Mplus Version 8.1).

## Discussion

This is the first RCT that aims to evaluate the effectiveness of a secondary low-intensity SoC and MI-based Chatbot-assisted intervention in reducing common digital addictions (including IGD and SMA), sedentary lifestyles, and mental distress (secondary outcomes) in at-risk adolescents.

We employ a psycho-educational approach as an active control group, which has significant implications for the internal validity of this trial. Unlike a “no-treatment” or “wait-list” control, an active control ensures that both groups receive an equivalent amount of attention and health information. This design allows the research team to control for non-specific therapeutic factors, such as the Hawthorne effect or simply the provision of health knowledge. Consequently, any observed superior outcomes in the intervention group can be more confidently attributed to the chatbot’s specific mechanisms of action, such as its MI- and SOC-based strategies, interactivity, personalization, and real-time positive reinforcement, rather than a mere increase in health awareness. From a public health perspective, this comparison is vital for determining whether digital interventions offer a more cost-effective and scalable alternative to traditional information-based methods. While the active control may narrow the observed effect size between groups, it enhances the clinical relevance of the findings, demonstrating whether the “what” and “how” of delivery are critical in modifying adolescent screen time related lifestyles and problems.

This is a comprehensive lifestyle promotion program for young people in the digital age. The anticipated findings from this RCT could have significant implications for public health strategies. Prolonged screen time and digital use problems are prevalent among local adolescents, while evidence-based treatment is largely scarce. Similar to other developing regions, HK has limited mental health professionals and long waiting times for mental health service [50]. Also, adolescents and their parents have low intention to seek professional help due to public stigma [51]. This study on an evidence-based online psychological intervention will benefit adolescents because psychotherapy is always preferred over pharmacotherapy for treating children/adolescents’ mental disorders and behavioral addictions, due to its higher efficacy, lower risk of being stigmatized, lower side-effects on children’s development, brain, and health, and higher preference by parents and children [52]. By demonstrating the effectiveness of the low-intensity Chatbot intervention, this RCT will provide a standardized, scalable, and sustainable intervention that can be integrated into routine social work practice and health system, specifically targeting at-risk populations. The use of Chatbot for 24/7 continuous health monitoring, coupled with personalized HC, may offer an expandable and sustainable approach to promoting healthy lifestyle changes. This could reduce the burden on healthcare systems by preventing future severe mental disorders and sedentary lifestyle-related physical problems (e.g., obesity). This meets the delivery of low-intensity interventions under the stepped care model, and can potentially have large health and economic impact. In the future, a cost-effectiveness analysis may be conducted to estimate the reduction in the healthcare burden arose from preventing severe mental and physical problems. Future studies could also explore strategies to enhance adherence and assess the long-term sustainability of Chatbot interventions.

## Supplementary Information

Below is the link to the electronic supplementary material.

Supplementary Material 1

Supplementary Material 2

Supplementary Material 3

## Data Availability

No datasets were generated or analysed during the current study.
