# Supplementary material for: Effects of low-intensity secondary theory-based Chatbot intervention for adolescents with digital addictions: protocol for a randomized controlled trial
Source: BMC Psychiatry. 2026 Jul 27;26:828. doi: 10.1186/s12888-026-08375-5 (PMC13712097; doi:10.1186/s12888-026-08375-5)
Supplement: Supplementary file 1 [file 12888_2026_8375_MOESM1_ESM.docx]

Appendix 1. Structural equation modeling for testing the mechanisms/mediators between the intervention group status and health outcomes

**Post-test**

**Primary outcomes:**

Composite outcome of internet gaming disorder and social media addiction

e

e

Intervention group status

e

**3-month follow-up**

**Primary outcomes:**

Composite outcome of internet gaming disorder and social media addiction

e

Autonomy

Competence

The hypothesized directionality of the relationships among the constructs and the overall fit of the model will be examined in the SEM-based path analysis. To test how well the model will fit the observed data, the χ^2^ test, the Comparative Fit Index (CFI), the non-normed fit index (NNFI), and the Root Means Square Error of Approximation (RMSEA) will be used. A non-significant p value (*p* > .05) of the χ^2^ test represents an adequate model fit. CFI and NNFI values of larger than .90 and a RMSEA value less than .08 indicate an acceptable model fit (1). Standardized path coefficients will be reported and compared by the chi-square difference test. Bootstrapping analyses (2) will be performed to identify the mediation effects of problem solving and time management for the relationships between intervention and outcomes. The 95% confidence intervals (CI) of the indirect effects will be obtained from 5,000 bootstrap resamples. A statistically significant mediation effect would be observed when the CI did not include zero.

References

1.	Hu Lt, Bentler PM. Cutoff criteria for fit indexes in covariance structure analysis: Conventional criteria versus new alternatives. Structural Equation Modeling: A Multidisciplinary Journal. 1999;6(1):1-55.

2.	Shrout PE, Bolger N. Mediation in experimental and nonexperimental studies: new procedures and recommendations. Psychological methods. 2002;7(4):422-5.
