# Supplementary material for: Effects of low-intensity secondary theory-based Chatbot intervention for adolescents with digital addictions: protocol for a randomized controlled trial
Source: BMC Psychiatry. 2026 Jul 27;26:828. doi: 10.1186/s12888-026-08375-5 (PMC13712097; doi:10.1186/s12888-026-08375-5)
Supplement: Supplementary file 2 [file 12888_2026_8375_MOESM2_ESM.docx]

Appendix 2. Details of intervention

**Rules based on stage of change**

Dichotomous or categorical classification of the participants into stages with regard to having more self-regulation for Internet gaming/social media use or reduce time on Internet games/social media by asking simple SoC questions (1, 2).

Are you planning to reduce time for Internet games/social media (or gain more control) in the next 7 days?

**P**

**yes**

**no**

**C**

Are you planning to reduce time for Internet games/social media (or gain more control) in the next 30 days?

**no**

**yes**

**PC**

Are you seriously considering having more self-regulation regarding Internet gaming/social media use?

or Do you have intention to reduce time on Internet games/social media?

**no**

**yes**

Do you successfully carry out your plan to regulate Internet gaming/social media use more than 30 days?

**no**

**yes**

**M**

**A**

**Note:** Pre-Contemplation (PC); Contemplation (C); Preparation (P); Action (A); Maintenance & relapse (M). The dichotomous classification assigns people, firstly, on the basis of whether they (yes or no) engage or not engage (yet) in the desired behavior. Secondly, for those who do not engage in the behavior, the intention to start the desired behavior within a certain time period is used to assign people to the precontemplation, contemplation, or preparation stage. Thirdly, for people who already engage in the desired behavior, the duration of the behavior is used to assign people to the action or maintenance stage.

**Intervention development**

Our intervention will be informed by: (1) focus group discussions, (2) a literature review, (3) expert discussions among the PI and co-Is based on their prior experience with MI- and SoC-based interventions, and (4) a pilot study.

First, focus group discussions with ten young people will explore motives for gaming and social media use, high-risk times and situations, perceived benefits and harms, motives and preferred strategies for self-regulation and behavioral management, alternative physical and social activities, and preferred avatar appearance.

Second, a comprehensive literature review will summarize existing findings on these issues. Together with focus group results, this will guide the development of intervention content and materials, including videos, gamification, metaphors, daily examples, experiential exercises, and narrative stories.

Third, the PI and co-Is have extensive experience in delivering MI- and SoC-based interventions for youth health behaviors (e.g., internet gaming and other addictions). Regular meetings will address design and implementation. MI experts will finalize intervention materials, and behavioral change experts will finalize SoC-based questions and logic.

Finally, a two-week pilot run with ten young people will collect feedback and suggestions to refine intervention content and implementation. The duration and logic will be slightly adjusted and finalized based on their input.

**Module plan**

The module plan will be developed based on previous SoC and MI-based in-person interventions for Internet addiction and Chatbot research (3, 4) and will be consolidated by our team. According to our previous studies in youth, over 70% endorsed self-diagnosed IGD; but self-corrective intention for Internet use-related problems ranged only from 28.2% to 34.1%; less than 40% perceived resources of help-seeking, and most perceived shame for help seeking; less than 40% intended to seek help from professionals (5, 6). The SoC thus comprehensively covered these different stages of change for digital use problems. The participants will be stage-matched in each module.

| Modules based on SoC | Outline and contents | Expected changes |
| --- | --- | --- |
| Pre-Contemplation (PC) | •Chatbot orientation and warm-up to build up rapport between the avatar (Virtual/e-Coach) and the participant. Chatbots will give a few lines to welcome the participant and politely ask permission for the next step.  •Monitoring stage of change by asking simple SoC questions.  •If the participant is assigned to this module based on his/her answer to the SoC questions, short MI pre-treatment videos ‘Planning for Change’ based on previous MI intervention studies (7): the videos will be designed using a positive, supportive, and empathetic style of language and contents without pressure. Chatbots will also interact with the participant to help his/her recognize the benefits and harms of internet gaming and/or social media using and the importance of healthy lifestyles.  •Chatbots will provide participants with a choice by seeking permission before providing the next video or further information in adherence to the MI principle.  •Common asked questions and responses  •Contact with real MI Coach if necessary | ↑Intrinsic motivation to engage in the intervention  ↑Awareness of negative consequences of digital addictions  ↑Intention to reduce digital addictions |
| Contemplation (C) | •Chatbot orientation and warm-up to build up rapport between the avatar (Virtual/e-Coach) and the participant. Chatbots will give a few lines to welcome the participant and politely ask permission for the next step.  •Monitoring stage of change by asking simple SoC questions.  •If the participant is assigned to this module based on his/her answer to the SoC questions, he/she will receive the package of videos and interactions to highlight the pros and cons of enhancing self-regulation of digital application use (5). It will help the participant to construct decisional balance about change and non-change which is in favor of changing current digital use behavior.  •The module will enhance the participant’s motivation and self-efficacy to facilitate possible changes via the lively narrative stories of peers who successfully recovered from internet gaming disorder and/or social media addiction.  •Common asked questions and responses  •Contact with real MI Coach if necessary | ↑Intrinsic motivation of behavior change  ↑Perceived autonomy, competence  ↑Behavior change intention |
| Preparation (P) | •Chatbot orientation and warm-up to build up rapport between the avatar (Virtual/e-Coach) and the participant. Chatbots will give a few lines to welcome the participant and politely ask permission for the next step.  •Monitoring stage of change by asking simple SoC questions.  •If the participant is assigned to this module based on his/her answer to the SoC questions, she/he will be provided with options to develop their own plans for changing digital use behavior, including digital use timetable and short-term and long-term goals and plans about study and future career. The goals will be developed based on the principles of Specific, Measurable, Achievable, Relevant, and Time-Bound (i.e., SMART goal).  •Various resources of support (e.g., the Youth Pre-Employment Training Program) will be pushed to the participant whenever appropriate.  •Information and encouraging messages to join exercises, interest classes or voluntary services will be pushed to the participant.  •Common asked questions and responses  •Contact with real MI Coach if necessary | ↑Cue to action  ↑Intrinsic motivation of behavior change  ↑Perceived autonomy, competence  ↑Behavior change intention |
| Action (A) | •Chatbot orientation and warm-up to build up rapport between the avatar (Virtual/e-Coach) and the participant. Chatbots will give a few lines to welcome the participant and politely ask permission for the next step.  •Monitoring stage of change by asking simple SoC questions.  •If the participant is assigned to this module based on his/her answer to the SoC questions, he/she will receive the package to strengthen his/her problem-solving skills in order to cope with the difficulties in implementing the action plan.  •Providing support and resources to enhance the participant’s self-efficacy in taking and maintaining the action.  •Expressing empathy for those facing difficulties during the changing process.  •Common asked questions and responses  •Contact with real MI Coach if necessary | ↑Cue to action  ↑Perceived autonomy, competence  ↑Quality of life, eHealth literacy techniques  ↓Sedentary lifestyle, mental distress |
| Maintenance & relapse (M) | •Chatbot orientation and warm-up to build up rapport between the avatar (Virtual/e-Coach) and the participant. Chatbots will give a few lines to welcome the participant and politely ask permission for the next step.  •Monitoring stage of change by asking simple SoC questions.  •If the participant is assigned to this module based on his/her answer to the SoC questions, he/she will receive the package that aims to identify the situations that may trigger relapse and to enhance the related coping strategies.  •Through interactions between Chatbots and the participant, the most important factors associated with the risk of relapse of the participant and the most effective coping strategies to modify the factors will be identified.  •Online resources for relapse-prevention techniques.  •Common asked questions and responses  •Contact with real MI coach if necessary | ↑Relapse-prevention and maintenance techniques  ↑Perceived autonomy, competence  ↑Quality of life, eHealth literacy techniques  ↓Sedentary lifestyle, mental distress |

**Chatbot system**

Figure 1. Architecture and basic workflow of the intelligent Chatbot for reducing common digital addictions, sedentary behaviors, and mental distress among adolescents.

Figure 1 illustrates the architecture and basic workflow of the intelligent Chatbot. Users interact with the Chatbot via instant messaging clients like WhatsApp. The Chatbot administers stage-targeted questionnaires, responds to queries, and delivers interventions based on stages of change and motivational interviewing outcomes. It tracks progress, monitors conversations, and sends automatic reminders. In early development, it can also consult human experts to ensure service quality.

The system comprises six modules: dialogue manager, logging system, Chatbot intelligence, quality assurance, data analytics, and database. This hybrid system combines rule-based logic with AI, enabling the Chatbot to respond to specific inputs while using natural language understanding (NLU) to identify user intents and entities.

Dialogue Manager (DM): Connects the Chatbot to WhatsApp and manages dialogue context. It receives user queries, extracts intents/entities via NLU, determines the best response, and replies to the user. Its three subcomponents—Dialogue State Tracker (DST), Dialogue Policy (DP), and Actions—maintain conversation state, decide the next step (e.g., follow-up question, suggestion, or confirmation), and execute Chatbot functions.

Chatbot Intelligence: Integrates NLU (trained on user inputs to return intents/entities), a knowledge base (for FAQs and domain-specific information), and expert-defined SoC rules. Additional services can be easily added.

Logging System: Records all conversation actions, performance metrics, and errors. Logs are essential for debugging, understanding user behavior, and system improvement.

Data Analytics: Performs real-time statistical analysis to monitor individual user progress, enable personalized features, and send automatic reminders based on user profiles.

Quality Assurance: Provides tools to build, train, test, and deploy new features. Conversation logs and feedback data help track performance, improve interactions, and retrain AI models.

Database: Identifies structured (e.g., SQL-stored conversational data) and unstructured (e.g., documents, multimedia) data sources, enabling intelligent interactions and providing stage-specific materials.

References

1.	Etter J-F, Perneger TV. A comparison of two measures of stage of change for smoking cessation. Addiction. 1999;94(12):1881-9.

2.	Dijkstra A. The Validity of the Stages of Change Model in the Adoption of the Self-Management Approach in Chronic Pain. The Clinical Journal of Pain. 2005;21(1):27-37.

3.	FHKPS B, Leung H. Development of an integrated intervention model for Internet addiction in Hong Kong. International Journal of Child and Adolescent Health. 2013;6(4):475.

4.	Cacanindin NM. Greening Food Consumption Using Chatbots as Behavioral Change Agent. Journal of Advanced Research in Dynamical and Control Systems. 2020;Vol. 12, 01(Special Issue).

5.	Lau JTF, Wu AMS, Cheng K-m, Tse VWS, Lau MMC, Yang X. Prevalence and factors of self-corrective intention among Hong Kong secondary school students who are self-assessed Internet addiction cases. Child and Adolescent Mental Health. 2018;23(3):155-63.

6.	Yang X, Ding N, Tu X, Zhao C, Xu H, Lai X, et al. The role of illness representations of internet gaming disorder (IGD) in help-seeking intention among undergraduates: a longitudinal study. Addictive Behaviors. 2022;128:107233.

7.	Soucy JN, Hadjistavropoulos HD, Karin E, Dear BF, Titov N. Brief online motivational interviewing pre-treatment intervention for enhancing internet-delivered cognitive behaviour therapy: A randomized controlled trial. Internet Interventions. 2021;25:100394.
