# Supplementary material for: Effects of low-intensity secondary theory-based Chatbot intervention for adolescents with digital addictions: protocol for a randomized controlled trial
Source: BMC Psychiatry. 2026 Jul 27;26:828. doi: 10.1186/s12888-026-08375-5 (PMC13712097; doi:10.1186/s12888-026-08375-5)
Supplement: Supplementary file 3 [file 12888_2026_8375_MOESM3_ESM.docx]

Appendix 3. Flow chart of the procedure

Post-intervention evaluation (T1)

3-month follow-up evaluation (T2)

SoC MI-based Chatbot group

Psycho-education control group

Randomization & baseline evaluation (T0)

School-based screening (presence of IGD/SMA) & participant invitation

Post-intervention evaluation (T1)

3-month follow-up evaluation (T2)

School invitation

Exclusion criteria: 1) currently taking psychotropic medication.
